# Epidemiology of transthyretin-associated familial amyloid polyneuropathy in the Majorcan area: Son Llàtzer Hospital descriptive study

**DOI:** 10.1186/1750-1172-9-29

**Published:** 2014-02-26

**Authors:** Juan Buades Reinés, Tomás Ripoll Vera, Mercedes Uson Martín, Hernán Andréu Serra, Ma Margarita Company Campins, José Miguel Diéguez Millán, Cristina Gallego Lezaun, Manuel Raya Cruz

**Affiliations:** 1 Internal Medicine, Son Llàtzer Hospital, Ctra. Manacor, Km 4, 0 07198 Palma de Mallorca Illes Balears, Spain; 2 Cardiology, Son Llàtzer Hospital, Mallorca, Spain; 3 Neurology, Son Llàtzer Hospital, Mallorca, Spain; 4 Digestive Medicine, Son Llàtzer Hospital, Mallorca, Spain; 5 Phatholoy Medicine, Son Llàtzer Hospital, Mallorca, Spain; 6 Ophalmology, Son Llàtzer Hospital, Mallorca, Spain

**Keywords:** Familial amyloid polyneuropathy, TTR, Amyloidosis, Transthyretin, Majorca, Asymptomatic carriers, Symptomatic patient, Liver transplant

## Abstract

**Background:**

Transthyretin-associated Familial Amyloid Polyneuropathy (TTR-FAP) is an autosomal dominant disease caused by the deposition of abnormal transthyretin that results from a gene mutation. Although rare worldwide, there are descriptions of several endemic foci, such as in Majorca, Balearic Islands, Spain. We aimed at describing a contemporary series of TTR-FAP patients in Son Llàtzer Hospital in Majorca from an epidemiological point of view in order to report their main clinical and laboratory characteristics.

**Methods:**

A retrospective, observational study was performed. Medical records from adult patients diagnosed with TTR-FAP from a single hospital were reviewed.

**Results:**

Out of a total of 107 cases, 75 subjects were included: asymptomatic carriers (52.3%) and symptomatic patients (47.7%). Mean age was 58.3 years at the time of the study, and 50.7% were men. Mean age at diagnosis was 49.8 years. In addition, 42 patients (39.2%) had received a liver transplant, and time to liver transplantation was on average 29.56 months from the initial diagnosis. They all had the Val30Met mutation. The organs of the nervous system were those most frequently impaired (57.3%), and 83.9% of the symptomatic patients were fully ambulant (stage 1). Family history was reported in 69.3% of the cases, with the patient’s father being the most commonly affected relative. Comorbidities were frequent, with high blood pressure being the most common.

**Conclusions:**

Our findings provide additional information on this condition and are useful for describing the demographic features, clinical presentation, diagnosis, and natural course of TTR-FAP in Majorca.

## Background

Familial amyloid polyneuropathies (FAP) are a group of systemic amyloidoses caused by the extracellular deposition of insoluble amyloid fibrils. Three main types of FAP have been described, based on the abnormal amyloid precursor protein resulting from a gene mutation (transthyretin [TTR], apolipoprotein A-1, or gelsolin) [1]. FAP caused by a mutated TTR is the most common form, with the Val30Met mutation in the TTR gene being by far the most common variant (accounting for about 50% of cases worldwide). It is virtually the only one in some countries, such as Portugal and Sweden [2], although at present 113 amyloidogenic mutations have been described in the TTR gene [1-3]. The abnormal TTR protein is mainly produced in the liver and to a lesser extent in the choroid plexus and retina.

Transthyretin-associated familial amyloid polyneuropathy (TTR-FAP) is a life-threatening disease transmitted as an autosomal dominant trait. The most typical presentation of TTR-FAP is a progressive nerve length-dependent, sensory-motor polyneuropathy, which usually begins with loss of thermal and pain sensation in the feet and slowly ascends up the limbs. This is then followed by autonomic manifestations and motor dysfunction or impairment. The progression of the disease is usually described as going through 3 stages according to the symptoms and signs shown by the patients. Stage 1 is characterized by mild motor dysfunction/impairment of the lower extremities, moderate autonomic manifestations, no or minimum impairment in daily activities, and full ambulation. Stage 2 is characterized by mild-to-moderate motor dysfunction/impairment of the lower or upper extremities, mild autonomic manifestations, significant impairment in daily activities, and the need for help in ambulation. Lastly, stage 3 is described as severe motor dysfunction/impairment of the lower or upper extremities, severe autonomic manifestations, profound impairment in daily activities, and confinement to a wheelchair [4]. Diagnosis is based on family history, neurographic evidence of polyneuropathy, detection of mutated TTR or DNA in the blood, and identification of amyloid deposits in the tissues (mainly in intestinal, nerve, salivary gland or abdominal fat biopsies). The disease is occasionally diagnosed in sporadic cases with no known family history. Immunolabeling or mass spectroscopy-based proteomic analysis can be useful in identifying the amyloid type, but a TTR/DNA test is mandatory [1]. Early- and late-onset presentations have been described, with late-onset presentations showing a slower progression and fewer autonomic symptoms. TTR-FAP is usually fatal within 10 to 13 years of the first symptoms [5-7].

Liver transplantation provides a useful and specific therapy for TTR-FAP by allowing for the suppression of the main source of the mutant TTR; it prevents neuropathy progression in approximately 70% of cases with Val30Met mutation on a long-term basis [8,9].

In November 2011, the European Medicines Agency [10] approved tafamidis meglumine, a small molecule that stabilizes and inhibits the dissociation of circulating TTR tetramers, thus preventing the deposition and formation of the abnormal TTR fibrils, for TTR-FAP Val30Met patients with stage-1 polyneuropathy, and it seems to be very promising [11,12].

TTR-FAP was initially described by Andrade in 1952 in Póvoa de Varzim, Portugal [13], and has subsequently been reported in several other European countries such as Sweden [14,15], as well as in countries like Japan [16,17] and Brazil [18,19], among others. It is a relatively rare disease worldwide, although it has a relatively high prevalence in some specific areas. A prevalence of 151/100,000 inhabitants has been reported in some areas in northern Portugal (including Póvoa de Varzim, where the first cases were discovered) [20] and of 104/100,000 inhabitants in northern Sweden [14]. The reported prevalence in Japan is 1.5/100,000 [16], and data from Cyprus suggest a prevalence of 3.72/100,000 [21].

In Spain, a significant area was found to be Majorca in the Balearic Islands. According to Munar-Qués et al. [22,23], the rate of prevalence amounts to 5/100,000 inhabitants in Majorca and 1/100,000 in Minorca, also a Balearic Island. TTR-FAP has also been described in other areas in Spain, including Valverde del Camino (Huelva), the Safor area (Valencia), Barcelona, Cantabria [24-26], and Vigo (Galicia) [25]. However, the number of patients in these areas is low. In Majorca the first case was described in 1976; subsequently the diagnosis of TTR-FAP was also clinically detected in 15 additional patients [22]. The disease could have arrived in Majorca from Portugal as a founder mutation in the 13th or 14th centuries, or it could have appeared independently in Majorca, as well as in other parts of the world. In 2005, a series of 102 confirmed patients from Majorca was well described by Munar-Qués et al. [27]. According to their conclusions, TTR-FAP seems to now be a growing public health problem in the Balearic Islands owing to its relatively high prevalence, together with its significant social and clinical impact.

We aimed at describing a contemporary series of TTR-FAP patients followed at Son Llàtzer Hospital, Majorca, from an epidemiological point of view and at reporting their clinical and laboratory characteristics.

## Methods

A retrospective, observational study was performed. Medical records from all adult (≥ 18 years old) patients who met the following inclusion criteria: being diagnosed with TTR-FAP at Son Llàtzer Hospital (a 350-bed community center located in Palma de Majorca, Spain). The study period ran from January 1, 2001 to January 13, 2012, at which point the recorded data were reviewed. We described both the symptomatic patients and the asymptomatic carriers at the time of diagnosis. The cohort of TTR-FAP patients that underwent liver transplantation was also described.

Demographic details (age, sex, occupational status) and clinical data (family history, type of mutation, and disease stage) were obtained. The results were analyzed by comparing asymptomatic carriers to symptomatic patients. As supplemental data we also recorded health care resource utilization from and at diagnosis.

Diagnostic criteria included: presence of TTR mutation (both in symptomatic patients and asymptomatic carriers).

Additional tests were performed in symptomatic patients: electromyogram to confirm the presence of mixed polyneuropathy; tissue biopsy to detect amyloid deposits; echocardiogram (ECG) or Holter monitoring (24-hour ECG) when cardiac involvement was suspected; and lastly, eye exam when appropriate.

Data confidentiality was respected when decoupling personal data. The study was classified by the Spanish Agency of Medicines and Medical Devices and was approved by the Independent Ethics Committee of Son Llàtzer Hospital.

Statistical analysis: Pearson’s chi-squared test or Fisher’s exact test were used for categorical data. Continuous variables were analyzed using Student’s *t*-test or one-way analysis of variance (ANOVA) (or Mann–Whitney *U* test for non-parametric variables). A *p*-value of < 0.05 was used to show statistically significant associations. All statistical analyses were based on SPSS ver. 17.0 or later.

## Results

A total of 107 cases with heterozygote TTR-Val30Met pathogenic mutations were diagnosed with TTR-FAP at Son Llàtzer Hospital in the study period running from January 2001 to January 2012. We have epidemiological and clinical data from 75 cases of asymptomatic carriers and symptomatic patients that were included in this study, and the remaining 42 patients received a liver transplantation.

### Description of asymptomatic carriers and symptomatic patients

**Figure 1:**
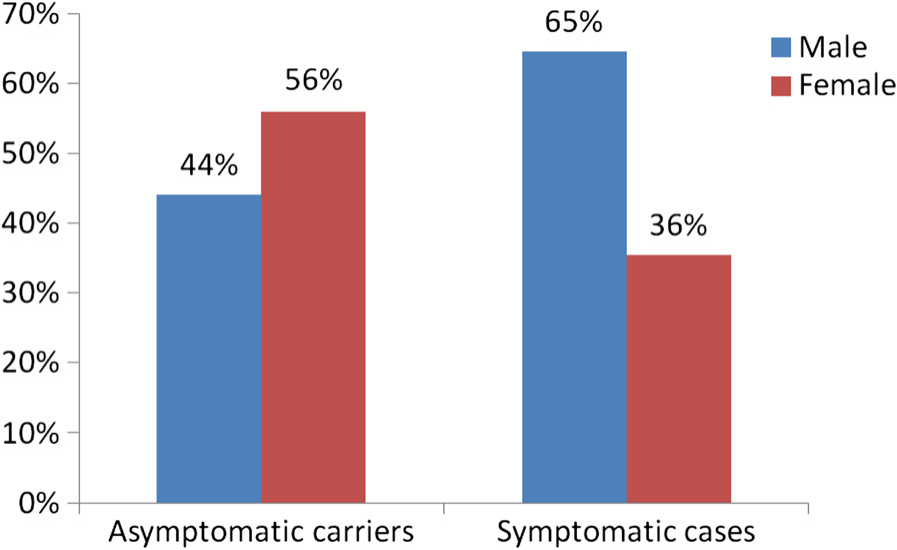
TTR-FAP related in Son Llàtzer Hospital (Majorca) comparison of asymptomatic carriers and symptomatic patients.

All of the subjects had the Val30Met mutation. Mean age was 58.33 ± 16.13 years (range from 28 to 92), and 38 (50.7%) were men (Figure 1) with a mean age at diagnosis of 49.8 ± 17.33 years (range from 13 to 81). Mean life expectancy without liver transplantation was 7.4 years. When assessing for this analysis (2011), we did not report all data, but 36 of 57 subjects (63.2%) had an active employment status. The rest of the demographic data are shown in Table 1.

**Table 1:** Demographic data of subjects included in the study at TTR-FAP diagnosis

**Variable**	Number*	**Percentage**	**Mean ± SD**	**Range**
**Sex (n = 75)**				
Men	38	50.7		
Women	37	49.3		
**Age, years (n = 75)**			58.33 ± 16.13	28–92
**Occupational status (n = 57)**				
Active	36	63.2		
Retired	16	28.1		
Other	5	8.8		
**Weight, kg (n = 24)**			67.00 ± 13.05	35–87
**Mean age at diagnosis, years (n = 54)**			49.8 ± 17.33	13–81

*Total number of patients analyzed; some patients did not report all data.

**Figure 2:**
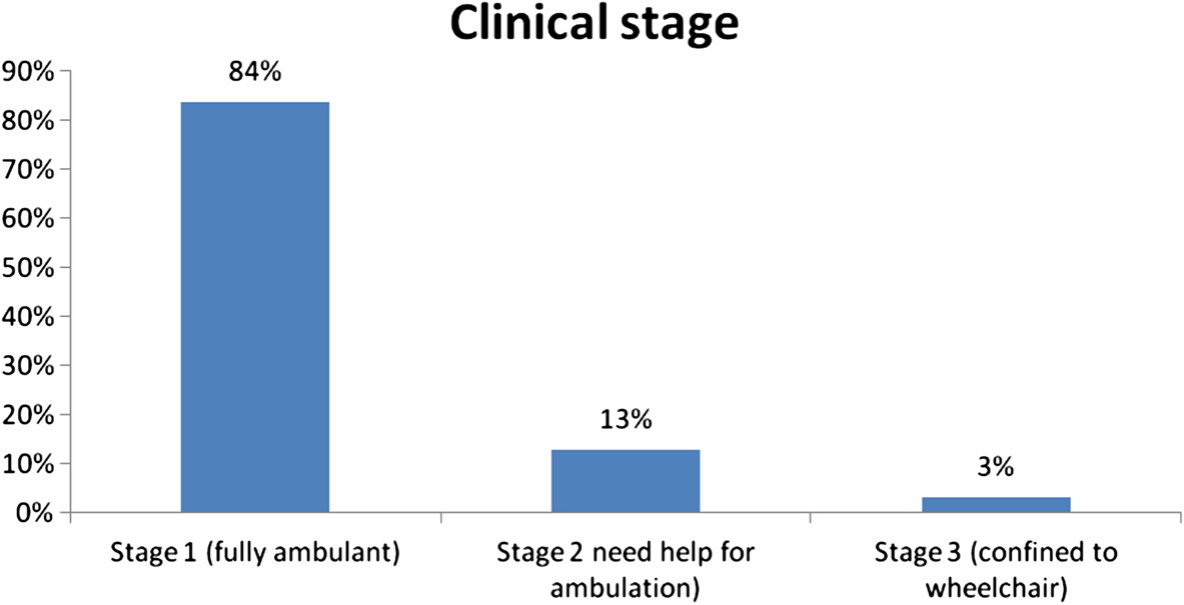
Clinical stage in symptomatic patients at diagnosis.

At diagnosis (we have data for 65 subjects), there were 34 (52.3%) asymptomatic carriers and 31 (47.7%) symptomatic patients. Clinical stage for those symptomatic patients at diagnosis was as follows: stage 1 (fully ambulant) for 26 patients (83.9%); stage 2 (needing help with ambulation) for 4 patients (12.9%); and stage 3 (confined to wheelchair) for 1 patient (3.2%) (Figure 2).

A family tree following the disease was performed for all patients and we found a family history of TTR-FAP in 52 cases (69.3%), with subjects’ fathers being the most commonly affected relative. Comorbidities at initial diagnosis were frequent 23 (30.7%), with high blood pressure being the most common, found in 18 subjects (24.0%). The rest of clinical data, including specific family history, comorbidities, and initial organ involvement, are shown in Table 2.

**Table 2:** Clinical data of patients included in the study at TTR-FAP diagnosis

**Variable**	**Number n = 75**	**Percentage**
**Family history**		
No	8	10.7
Yes	52	69.3
Father	22	42.3
Mother	12	23.1
Grandfather/grandmother	3	5.8
Uncle/aunt	16	30.8
Sibling	23	44.2
Other (cousin, niece, nephew, etc.)	26	50.0
Unknown	15	20
**Comorbidities**		
Any comorbidity	23	30.7
High blood pressure	18	24.0
Dyslipidemia	7	9.3
Diabetes mellitus	3	4.0
Kidney failure	3	4.0
Smoking	3	4.0
Obesity	2	2.7
Heart failure	1	1.3
**Initial organ involvement**		
Nervous system	43	57.3
Gastrointestinal tract	11	14.7
Erectile dysfunction	4	5.3
Cardiovascular	3	4.0
Kidneys	3	4.0

**Figure 3:**
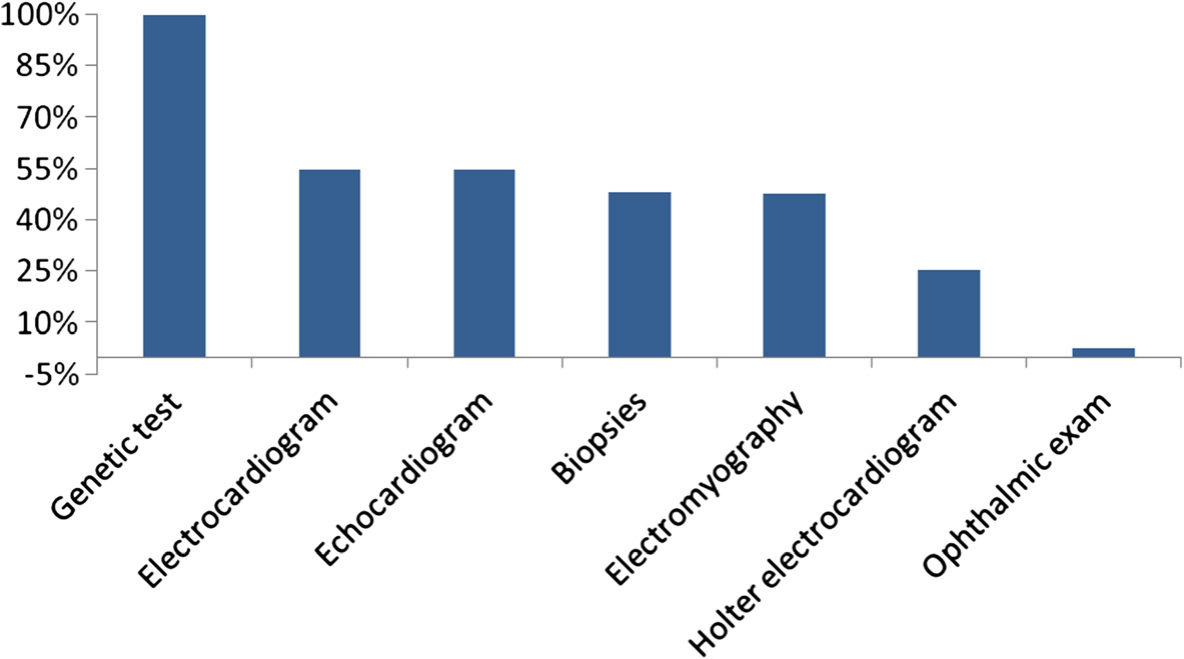
Additional tests performed.

All subjects were diagnosed with a TTR/DNA/genetic test. Moreover, additional tests were performed in symptomatic patients: electromyogram to rule out the presence of mixed polyneuropathy; biopsy to try to detect tissue amyloid deposits; echocardiogram or Holter monitoring (24-hour ECG) when cardiac involvement was suspected; and ophthalmic exam when appropriate (Figure 3).

In the comparison between asymptomatic carriers and symptomatic patients, we found statistically significant differences in mean age at diagnosis (54 vs. 44 years; *p* < 0.05) and also for active employment status (75.8% vs. 50.0%; *p* < 0.01). In contrast, we found no statistically significant differences in mean age. Comorbid disease profile was similar in both groups.

### Patients with liver transplantation

During the data analysis, liver transplantation had already been performed in 42 patients (39.2%). Not all the patients could undergo liver transplantation, mainly owing to advanced age, contraindication (comorbidities), or because they were at stage 3 of the disease progression. The time to inclusion on the transplantation waiting list was 29.56 months (range from 16.72 to 37.88).

After transplantation, 34 patients (80.9%) showed no disease progression; 23 (54.7%) needed no help with ambulation; and most patients (35, or 83.3%) stated that they were satisfied with their therapy (transplantation). The post-transplantation course was generally good, with a relatively high proportion of patients being stable and with many subjects having an active employment status (77.8% of patients were able to resume their usual working activity).

## Discussion

Our findings, which are based on a relatively large series from a local community hospital in Majorca, provide additional information on this condition and are useful for describing the demographic features, clinical presentation, diagnosis, and natural course of TTR-FAP in the Majorca area, a significant endemic focus.

**Figure 4:**
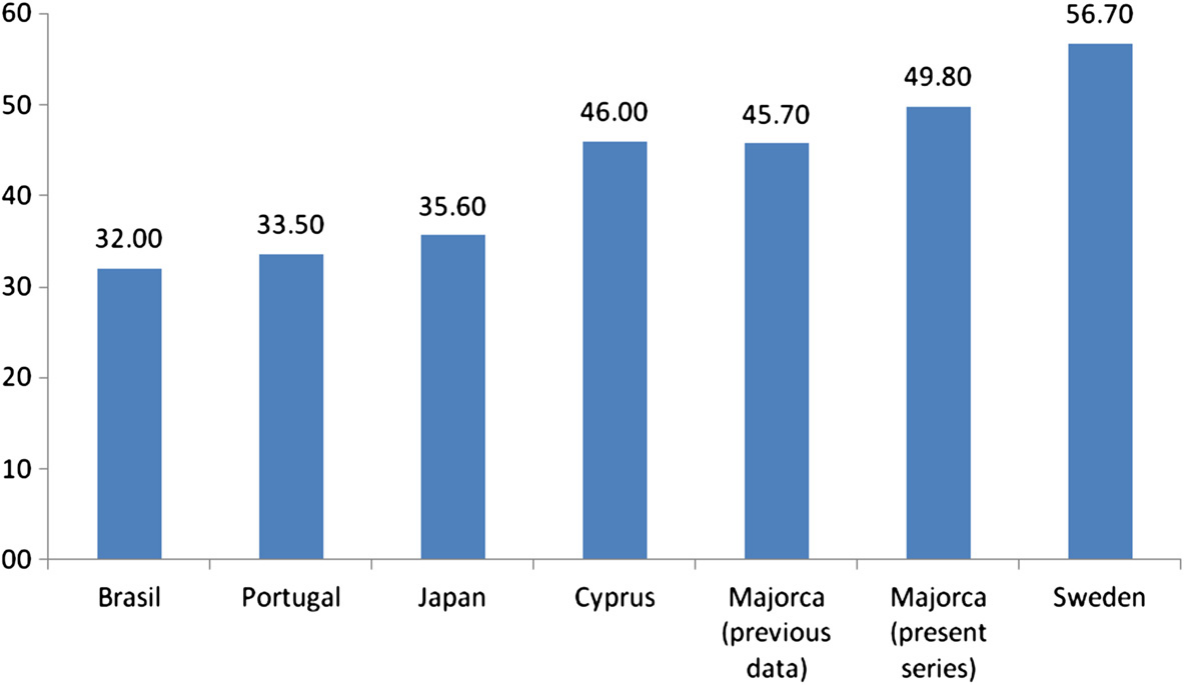
**Mean age of onset or diagnosis of TTR-FAP in several series **[14,16,20-22]**.**

Age of onset has not been consistent in all reported series (Figure 4). Our findings (mean age at diagnosis of 49.8 years) are similar to previously reported data from Majorca (age of onset of 45.7 years) [27], as well as to those from some other Mediterranean areas, like Cyprus (46 years) [21]. An earlier age of onset has been found in the main endemic areas in Portugal (33.5 years) [20], Japan (35.6 years) [16], and Brazil (32 years) [18], whereas a later age of onset has been reported in northern Sweden (56.7 years) [15]. In contrast, an even later age of onset (61 years) has been reported in sporadic cases in a series from a non-endemic area [28]. Taking into account that the causal mutation is probably the same in Portugal and Majorca, and that patients who are not aware of relatives who had the disease (sporadic cases) in other areas show a later age of onset, the difference in age of onset between Portuguese and Majorcan cases could possibly be owing to environmental or epigenetic factors.

Although a male predominance has been previously reported in Majorca (62% versus 38%) [27], our results show a ratio of 51% to 49%. Similar findings have been reported in some other areas, including Brazil (45% to 55%) [18] and Cyprus (44% to 56%) [21]. A later age of onset in women has been described in Portugal [20] and Brazil [18], whereas no differences based on gender were found in Sweden [15], Cyprus [21], or the previous Majorcan series [27]. Our findings confirm a similar age of onset in both men and women.

Clinical data from our series show a high proportion of patients with a positive family history, mostly owing to the father. As expected, the existence of a family history was more common in asymptomatic carriers. Such individuals had also an earlier diagnosis. A large number of symptomatic patients received a liver transplant, with mean time to receiving a liver transplant being just slightly longer than 2.5 years from the patient’s diagnosis. Data on employment activity are scarce in previous studies, and our findings are particularly relevant for demonstrating the real impact of the disease on a patient’s life.

### Weaknesses

This is the first TTR-FAP epidemiological study to include both clinical and work productivity data during disease progression. The isolated nature of TTR-FAP and its being limited to a specific geographical area make it difficult to extrapolate results. In addition, limitations stemming from the retrospective nature of this study are also applicable.

## Conclusions

TTR-FAP is a life-threatening neurodegenerative disease that constitutes a public health problem in Majorca, with a disease focus growing mainly due to TTR-FAP is being better understood so patients could be diagnosed earlier. The clinical characteristics of our series are similar to those previously reported in the literature, especially in Mediterranean areas, but with a slightly older age at onset and relatively higher neurological and digestive involvement at presentation. Because TTR-FAP is a multi-pathological disease, in Majorca a multidisciplinary group has been created that includes experts in internal medicine, neurology, cardiology, gastroenterology, ophthalmology and pathology, thereby enabling a quicker assessment of disease involvement at presentation. This multidisciplinary approach will probably decrease the age of onset in Majorca in further studies. The proportion of symptomatic TTR-FAP patients in our series who underwent liver transplantation seems relatively high (56%) compared with other countries.

## Abbreviations

FAP: Familial amyloid polyneuropathy; TTR: Transthyretin; TTR-FAP: Transthyretin-associated familial amyloid polyneuropathy; ECG: Echocardiogram; ANOVA: Analysis of variance.

## Competing interests

This study was sponsored by Pfizer Spain. Editorial Support (medical writing) was provided by Content Ed Net Communications S.L. and was funded by Pfizer Spain.

## Authors’ contributions

All authors had complete access to the data, participated in the analysis and/or interpretation of results, and drafted the manuscript. All authors read and approved the final manuscript.

## References

[B1] Plante-Bordeneuve V Said G Familial amyloid polyneuropathy Lancet Neurol 2011 10 1086 1097 10.1016/S1474-4422(11)70246-0 22094129

[B2] Saraiva MJ Transthyretin mutations in hyperthyroxinemia and amyloid diseases Hum Mutat 2001 17 493 503 10.1002/humu.1132 11385707

[B3] Connors LH Lim A Prokaeva T Roskens VA Costello CE Tabulation of human transthyretin (TTR) variants Amyloid 2003 10 160 184 10.3109/13506120308998998 14640030

[B4] Coutinho P Silva AM Lima LJ Glenner GG Forty years of Eexperience with type I amyloid neuropathy. review of 483 cases Amyloid and Amyloidosis 1980 Amsterdam: Excerpta Medica 88 98

[B5] Said G Plante-Bordeneuve V Familial amyloid polyneuropathy: a clinico-pathologic study J Neurol Sci 2009 284 149 154 10.1016/j.jns.2009.05.001 19467548

[B6] Plante-Bordeneuve V Said G Transthyretin related familial amyloid polyneuropathy Curr Opin Neurol 2000 13 569 573 10.1097/00019052-200010000-00011 11073365

[B7] Suhr O Danielsson A Holmgren G Steen L Malnutrition and gastrointestinal dysfunction as prognostic factors for survival in familial amyloidotic polyneuropathy J Intern Med 1994 235 479 485 10.1111/j.1365-2796.1994.tb01106.x 8182405

[B8] Sharma P Perri RE Sirven JE Zeldenrust SR Brandhagen DJ Rosen CB Douglas DD Mulligan DC Rakela J Wiesner RH Balan V Outcome of liver transplantation for familial amyloidotic polyneuropathy Liver Transpl 2003 9 1273 1280 10.1016/j.lts.2003.09.016 14625827

[B9] Adams D Samuel D Slama M Treatment of familial amyloid polyneuropathy Presse Med 2012 41 793 806 10.1016/j.lpm.2011.11.027 22341949

[B10] European Medicines Agency Vyndaqel. Summary of product characteristics [ http://www.ema.europa.eu/docs/en_GB/document_library/EPAR_-_Product_Information/human/002294/WC500117862.pdf]

[B11] Said G Grippon S Kirkpatrick P Tafamidis Nat Rev Drug Discov 2012 11 185 10.1038/nrd3675 22378262

[B12] Russo M Stancanelli C Gentile L Toscano A Vita G Mazzeo A Safety and tolerability of orally administered tafamidis meglumine in TTR FAP: preliminary data at 3-month follow-up J Peripher Nerv Syst 2012 17 S48 S49

[B13] Andrade C A peculiar form of peripheral neuropathy; familiar atypical generalized amyloidosis with special involvement of the peripheral nerves Brain 1952 75 408 427 10.1093/brain/75.3.408 12978172

[B14] Andersson R Familial amyloidosis with polyneuropathy. A clinical study based on patients living in northern Sweden Acta Med Scand Suppl 1976 590 1 64 1064291

[B15] Sousa A Andersson R Drugge U Holmgren G Sandgren O Familial amyloidotic polyneuropathy in Sweden: geographical distribution, age of onset, and prevalence Hum Hered 1993 43 288 294 10.1159/000154146 8406517

[B16] Araki S Mawatari S Ohta M Nakajima A Kuroiwa Y Polyneuritic amyloidosis in a Japanese family Arch Neurol 1968 18 593 602 10.1001/archneur.1968.00470360015001 5652991

[B17] Ikeda S Nakazato M Ando Y Sobue G Familial transthyretin-type amyloid polyneuropathy in Japan: clinical and genetic heterogeneity Neurology 2002 58 1001 1007 10.1212/WNL.58.7.1001 11940682

[B18] Bittencourt PL Couto CA Clemente C Farias AQ Palacios SA Mies S Goldberg AC Phenotypic expression of familial amyloid polyneuropathy in Brazil Eur J Neurol 2005 12 289 293 10.1111/j.1468-1331.2004.00941.x 15804246

[B19] Saporta MA Zaros C Cruz MW Andre C Misrahi M Bonaiti-Pellie C Plante-Bordeneuve V Penetrance estimation of TTR familial amyloid polyneuropathy (type I) in Brazilian families Eur J Neurol 2009 16 337 341 10.1111/j.1468-1331.2008.02429.x 19364362

[B20] Sousa A Coelho T Barros J Sequeiros J Genetic epidemiology of familial amyloidotic polyneuropathy (FAP)-type I in Povoa do Varzim and Vila do Conde (north of Portugal) Am J Med Genet 1995 60 512 521 10.1002/ajmg.1320600606 8825887

[B21] Dardiotis E Koutsou P Papanicolaou EZ Vonta I Kladi A Vassilopoulos D Hadjigeorgiou G Christodoulou K Kyriakides T Epidemiological, clinical and genetic study of familial amyloidotic polyneuropathy in Cyprus Amyloid 2009 16 32 37 10.1080/13506120802676948 19291512

[B22] Munar-Ques M Costa PP Saraiva MJ Viader-Farre C Munar-Bernat C Familial type I (Portuguese form) amyloidotic polyneuropathy in Majorca. Study using the TTR (Met30) genetic marker Med Clin (Barc) 1988 91 441 444 3210815

[B23] Munar-Ques M Familial amyloid polyneuropathy 2003 Med Clin (Barc) 2003 121 100 101 10.1016/S0025-7753(03)73869-X 12855135

[B24] Munar-Ques M Up-date on amyloidosis. Hereditary amyloidosis Med Clin (Barc) 1994 103 109 115 8065215

[B25] Tornero Estebanez C Soriano Soriano C Gimenez Escrich A Rull Segura S Late-onset familial amyloid polyneuropathy in the Safor (Valencia) area: four case reports Rev Clin Esp 2007 207 75 76 10.1157/13100199 17397566

[B26] Gómez H Araujo-Fernández S Arca-Blanco A Novoa-Lamazares L González-Vázquez L Sánchez-Conde P Durán Muñoz O De La Fuente Aguado J Servicio de Medicina Interna. Hospital Povisa. Vigo. Polineuropatía amiloidótica familiar tipo 1 de Andrade: serie de 4 casos 2011 Ferrol, Spain: In XXVIII Reunión de la Sociedad Gallega de Medicina Interna 13-14 May

[B27] Munar-Ques M Saraiva MJ Viader-Farre C Zabay-Becerril JM Mulet-Ferrer J Genetic epidemiology of familial amyloid polyneuropathy in the Balearic Islands (Spain) Amyloid 2005 12 54 61 10.1080/13506120500032741 16076612

[B28] Plante-Bordeneuve V Ferreira A Lalu T Zaros C Lacroix C Adams D Said G Diagnostic pitfalls in sporadic transthyretin familial amyloid polyneuropathy (TTR-FAP) Neurology 2007 13 693 698 17698792 10.1212/01.wnl.0000267338.45673.f4

